# Sorbin and SH3 domain-containing protein 2 (SORBS2) is a component of the acto-myosin ring at the apical junctional complex in epithelial cells

**DOI:** 10.1371/journal.pone.0185448

**Published:** 2017-09-29

**Authors:** Karin Fredriksson-Lidman, Christina M. Van Itallie, Amber J. Tietgens, James M. Anderson

**Affiliations:** Laboratory of Tight Junction Structure and Function, National Heart Lung and Blood Institute, National Institutes of Health, Bethesda, Maryland, United States of America; Emory University School of Medicine, UNITED STATES

## Abstract

SORBS2 is a scaffolding protein associated with Abl/Arg non-receptor tyrosine kinase pathways and is known to interact with actin and several other cytoskeletal proteins in various cell types. Previous BioID proximity labeling of tight and adherens junction proteins suggested that SORBS2 is a component of the apical junction complex of epithelial cells. We asked whether SORBS2 plays a previously unappreciated role in controlling perijunctional actin and tight junction barrier function. Using super resolution imaging we confirmed that SORBS2 is localized at the apical junction complex but farther from the membrane than ZO-1 and located partially overlapping both the tight- and adherens junctions with a periodic concentration that alternates with myosin IIB in polarized epithelial cells. Overexpression of GFP-SORBS2 recruited alpha-actinin, vinculin and N-WASP, and possibly CIP4 to cellular junctions. However, CRISPR-Cas9 knock-out of SORBS2 did not alter the localization- or immunofluorescent staining intensity of these or several other junctional- and cytoskeletal proteins. SORBS2 knock-out also did not affect the barrier function as measured by TER and dextran flux; nor did it change actin-dependent junction re-assembly as measured by Ca^2+^-switch and Latrunculin-B wash-out assays. The kinetics of HGF-induced cell scattering and wound healing, and dextran flux increase induced by PDGF also were unaffected by SORBS2 knock-out. SORBS2 concentrates with apical junctional actin that accumulates in response to knock-down of ZO-1 and ZO-2. In spite of our finding that SORBS2 is clearly a component of the apical junction complex, it does not appear to be required for either normal tight- or adherens junction assembly, structure or function or for growth factor-mediated changes in tight junction dynamics.

## Introduction

Tight junctions (TJ) form a continuous apicolateral paracellular barrier between epithelial cells that enables selective and regulated movement of solutes between the apical and basolateral compartments. To date numerous proteins have been identified at the TJ [1], including the transmembrane claudin family of proteins [2–4], occludin [5] and the scaffolding proteins ZO-1, -2 and -3 [6], which connect the transmembrane proteins to the actin cytoskeleton. Despite the many identified TJ proteins there are most likely many more proteins that play important roles for TJ regulation and function. In an effort to find novel TJ interacting proteins, we previously utilized a proximity-dependent biotinylation method (BioID) [7], followed by proteomic analysis of biotinylated proteins neighboring ZO-1 and occludin [8,9]. Among the most abundant proteins recovered when an engineered biotin ligase was fused to the N-terminus of ZO-1 was the actin-binding and signaling protein Sorbin and SH3 domain-containing protein 2 (SORBS2, also called ArgBP2); it was not detected in a similar analysis of proteins proximal to the C-terminus of ZO-1 [9] (S1 Table). SORBS2 was also identified as proximal to both the N- and C-terminus of occludin [8] (S1 Table), although with relatively lower abundance. The selective identification of SORBS2 as close to the N- but not the C-terminus of ZO-1 raised the possibility that SORBS2 might have a functional spatially specific role in regulating properties of the TJ.

SORBS2 was originally characterized as an interactor of Arg kinase [10] and was subsequently identified as a member of a three-protein family, including CAP ((c-Cbl-associated protein)/ponsin/SORBS1) and vinexin (SORBS3) [11]. All three share an N-terminal sorbin homology domain (SoHo), which has been reported to interact with flotillin [12–14], and three C-terminal SH3 domains [11,15]. SORBS1, SORBS2 and SORBS3 localize at focal adhesions and SORBS1 and SORBS2 also localize at adherens junction based cell-cell contacts [15–20]. In addition, SORBS2 is also associated with actin stress fibers [10,19,21]. More relevantly, SORBS2 was previously co-localized with ZO-1 at tight junctions in murine epithelial NMuMG cells; however, a role for tight junction SORBS2 was not defined [19]. SORBS2 has been reported to negatively regulate Abl kinase [22], recruit Pyk2/Cbl complex to the lipid raft compartments [14] and bind to Akt [23]. In addition, evidence suggests that SORBS2 is also involved in cell adhesion, actin/cytoskeletal organization [10,24,25] and migration of tumor cells [17,25]. Together these observations raise the interesting possibility that SORBS2 might have a role in a signaling pathway regulating perijunctional actin, possibly through a tyrosine kinase pathway in epithelial cells.

In the present study, we sought to better describe the localization of SORBS2 relative to established epithelial tight and adherens junctions (AJ) proteins using super resolution microscopy and to explore a potential role at the TJ by functional analysis, both at baseline, and after growth factor treatment. We compared two control epithelial cell lines derived from two different tissues, namely human intestine and canine kidney, with the same cell lines in which SORBS2 had been deleted by CRISPR-Cas9. We used a wide range of standard assays to evaluate barrier properties and perijunctional actin regulation. We could show that SORBS2 is a component of the perijunctional actomyosin ring and it is both recruited to actin, and can concentrate actin when it is over-expressed, consistent with its being an actin binding protein. Its localization clearly overlaps both the TJ and AJ, but it does not appear to be essential either for normal tight- or adherens junction assembly, structure or function or for growth factor-mediated changes in tight junction dynamics.

## Materials and methods

### Cell culture, CRISPR-Cas9 knock-out and transfections

Madin-Darby Canine Kidney (MDCKII) epithelial cells (WT tet-off (TO) MDCKII cells, BD Biosciences, WT MDCKII cells (CCL34), American Type Culture Collection, Manassas, VA and ZO-1 and ZO-2 double knock-down TO MDCKII cells [26]) were cultured under standard culture conditions in DMEM (Mediatech, Manassas, VA; 4.5 g/l glucose), 1 x penicillin/streptomycin (Mediatech) and 10% tet-tested fetal calf serum (FCS; Atlanta Biological, Norcross, GA). ZO-1 and ZO-2 double knock-down cells were also cultured with the addition of doxycycline (50 μg/ml, Sigma-Aldrich, St. Louis, MO) for experiments to turn off the ZO-1 rescue construct. HEK 293 Tet-Off^®^ Advanced cell line (Clontech, Mountain View, CA) was cultures as described above. Human intestinal epithelial cell line SKco15, a kind gift from Dr. Asma Nusrat (University of Michigan), was cultured in the same media as MDCKII cells but with the addition of 10 mM 4-(2-hydroxyethyl)-1-piperazineethanesulfonic acid (HEPES, Mediatech), pH 7.4, and 1× nonessential amino acids (NEAA; Mediatech) and 10% FCS under normal culture conditions. Human recombinant HGF and PDGF-BB was obtained from PeproTech Inc (Rocky Hill, NJ). For all SKco15 experiments cells were grown on collagen coated surfaces (rat tail collagen, Sigma-Aldrich). Both MDCKII and SKco15 cells were passaged every 4–7 days. When cultured on Transwell filter inserts (Corning, Corning, NY) all cells were plated at a density of 0.5 x 10^5^/insert and cultured to confluency (usually within 48h) before transepithelial resistance (TER) was measured daily for seven days. When cells were used for further experiments such as dextran flux, immunofluorescent staining or immunoblots they were harvested at seven days after confluency (9 days after plating).

Stable SORBS2 knock-out (KO) cell lines were obtained by transfecting cells with pSpCas9(BB)-2A-Puro (PX459) V2.0 (62988; Addgene, Cambridge, MA; [27]) containing SORBS2 sgRNA (S2 Table) using Lipofectamine 2000. After selection for 48 h in 2 μg/ml puromycin (Life Technologies, Carlsbad, CA), dilution cloning into 96-well plates was performed to obtain single cell clones. Clonal lines were expanded and tested for SORBS2 expression 2–3 weeks (MDCKII) or 5–6 weeks (SKco15) after transfection with SORBS2 antibody by immunoblot. Genomic DNA was collected (DNeasy; Qiagen, Hilden, Germany) from cell lines negative for SORBS2 by immunoblot and PCR amplification (Phusion, HF kit; New England Biolabs, Ipswich, MA) of the region of interest was performed using specific SORBS2 primers (S2 Table). PCR product was loaded to a 1% agarose gel (SeaKem^®^GTG^®^ agarose; Lonza, Basel, Switzerland) and after electrophoresis the DNA bands were visualized with Ethidium bromide and UV light (Benchtop 2UV™ Transilluminator; UVP, Upland, CA), excised and gel purified (QIAquick®, Qiagen) before sending the DNA for sequencing to ACGT Inc (Germantown, MD). Sequencing primers used were the same as for the amplification (S2 Table).

Full length SORBS2 (pcDNA3-myc-ArgBP2α) was a generous gift from Dr. Koh-Ichi Nagata and was cloned by In-Fusion cloning with specific primers (S2 Table) into pEGFP-C1 vector (Clontech #6084–1; PT3028-5). EGFP-SORBS2 was transiently expressed in MDCKII cells after transfection with Lipofectamine 2000 (Life Technologies). Cells were fixed and stained for immunofluorescence 72 hours post transfection.

Full length SORBS1 (pcMV6-Entry-myc-DDK-SORBS1; RC224656 (NM_006434)) was obtained from OriGene (Rockville, MD). myc-DDK-SORBS1 was transfected using Lipofectamine 2000 (Life Technologies) and expressed transiently in HEK293 Tet-Off^®^ Advanced cells. Cell lysates were used to test specificity of the SORBS1 antibody.

### SORBS1, 2 and 3 qRT-PCR and SORBS2 PCR for isoform identification

Total RNA was collected using RNeasy (Qiagen) from WT and SORBS2 KO MDCKII cells grown on culture dishes until confluency. Reverse transcription was performed using SuperScript^®^ VILO™ kit (ThermoFisher, Waltham, MA). qRT-PCR was performed as previously described [28] using primers for canine SORBS1, SORBS2, SORBS3 and ZO-1 (SORBS1 and SORBS3 primers: S2 Table, ZO-1 primers: previously published [28]).

For SORBS2 isoform identification, mRNA was collected from WT SKco15 and MDCKII cells and reverse transcription was performed as described above. Primers were designed that could identify all, or specific isoforms, of SORBS2 in canine- or human cells (SORBS2 primers, S2 Table) and DNA was amplified (Phusion, HF kit, New England Biolabs). The PCR products were loaded to a 1% agarose gel (SeaKem^®^GTG^®^ agarose, Lonza) and separated by electrophoresis. Ethidium bromide-stained DNA bands were visualized by UV imaging (MyECL imager, ThermoFisher).

### Antibodies

A custom Rabbit anti-human SORBS2 C-terminal (7515) antibody was manufactured by Covance (Madison, WI) against the immunogen peptide sequence CSNKPQRPVFTHENIQ. Verification of the antibody was performed in a multi-step effort. Initial verification was performed by immunoblotting lysates from WT SKco15 cells and MDCKII cells, comparing signals for antibody pre-absorbed with the immunogen to those from untreated antibody. Secondly, antibody specificity was tested by immunoblot with SORBS2 KO cell lysates versus WT lysates and with lysates from HEK293 cells transfected with human GFP-SORBS2. Mouse anti-ZO-1, rat anti-E cadherin, mouse anti–α-actinin, mouse-anti-CIP4, mouse anti–γ-tubulin, rabbit anti–myosin 2B, rabbit anti-GFP, rabbit anti-N-WASP and mouse anti-occludin were described previously [28]. Rat anti-ZO-1(R40.76) [29], mouse anti-myosin2B (3H2, ab684; Abcam, Cambridge, MA), mouse anti-myosin2A (ab55456; Abcam), rabbit anti-myosin2A (909801, BioLegend, San Diego, CA), rabbit anti-cingulin (C532 was a generous gift from Dr. Sandra Citi, University of Geneva, Geneva, Switzerland), mouse anti-vinculin (h-VIN-1, V9131; Sigma-Aldrich), mouse anti-afadin (AF6; BD Transduction Laboratories, Franklin Lakes, NJ), mouse anti-p120 catenin (BD Transduction Laboratories), rabbit anti-SORBS1 (ProteinTech, Chicago, IL). Species-specific secondary antibodies for immunofluorescence (Cy2, Cy3, and Cy5 conjugated) as well as F(ab’)_2_-fragments used for super resolution microscopy (Alexa Fluor^®^ 647- and 594-conjugated) and immunoblots (IR-labeled 680/700 and 790/800 antibodies) were from Jackson ImmunoResearch (West Grove, PA). Rhodamine–phalloidin was from Life Technologies.

### Immunofluorescence microscopy

MDCKII cells were cultured on uncoated and SKco15 cells on collagen-coated Transwell filters (Corning), followed by fixation in either 1% paraformaldehyde (PFA) or 100% ice-cold ethanol as described previously[28]. Filters were mounted with Mowiol (EMD, Billerica, MA) containing 1% *n*-propyl gallate (Sigma-Aldrich). Frozen tissue sections from mouse liver were obtained from the National Heart, Lung, and Blood Institute Pathology Core; animal procedures were carried out in accordance with the guidelines of the National Heart, Lung, and Blood Institute Animal Care and Use Committee. Tissue sections were fixed in 1% PFA and immunofluorescence procedures carried out as described above. Fixed samples were imaged on a Zeiss (Thornwood, NY) 710 confocal microscope, using 63×/NA 1.4 oil objective or 20x air, with 488-, 561-, and 633-nm laser lines, or a Zeiss LSM 880 Airyscan in Super resolution mode with a 63x 1.4 NA objective. Raw data were processed using Airyscan processing with “auto strength” (Mean strength +/- S.D. = 5.5+/- 1.3) in Zen Black software version 2.3.

Super resolution imaging was performed using Stimulated Emission Depletion (STED) microscopy. STED images were obtained using a commercial Leica SP8 STED 3X system (Leica Microsystems, Mannheim, Germany), equipped with a white light excitation laser and 3 depletion lasers: 592, 660, and 775nm. A 100×/1.4 NA oil immersion objective lens (HC PL APO CS2, Leica Microsystems) was used for imaging. Image stacks were collected as 8 bit, 1,024 × 1,024 pixel images with 25 nm *x* and *y* pixel sizes and 0.2 μm *z*-steps. Pinhole was set to 0.7 AU, 6 line averages and frame accumulation = 2 (phalloidin) and 4 line averages and frame accumulation = 1 (everything else). Rhodamine phalloidin was imaged at 568 nm (emission bandwidth 578–737 nm, 775 STED pulsed depletion laser set at 50% power), 594-conjugated F(ab’) fragments was imaged at 594 nm (emission bandwidth 604–737 nm, 775 STED pulsed depletion laser set at 20% power) and 647-conjugated F(ab’) fragments at 647 nm (emission bandwidth 658–737 nm, 775 STED pulsed depletion laser set at 8% power); time gating was set to a range of 0.7–6.5 ns (nanoseconds). STED image stacks were deconvolved using Huygens software (Scientific Volume Imaging B.V., Hilversum, The Netherlands). ImageJ FIJI (NIH, Bethesda, MD) was used to make maximum intensity Z-projections of the total 2μm stacks imaged and to perform line scans of fluorescence intensity to confirm the visual observations. Twenty line scans/full image field were performed, each centered over cell contacts. Lines were 1000 nm in length and placed where SORBS2 could be found on both sides of the cell-cell junction, regardless of the fluorescence intensity on each side.

### Immunoblots

Immunoblots were performed with MDCKII or SKco15 cell lysates. Cells were grown either on Transwell filters (Corning) or in 24-well plates (Corning) for seven days after confluency. One hundred μl 4x SDS-sample buffer was added directly to each well, or filters were excised and placed directly in 100 μl 4x SDS-sample buffer. All samples were briefly sonicated before an equal volume was either frozen at -20°C until use or loaded on a SDS-PAGE gel (4–12% Bis-Tris, 1 mm thickness; NuPage™, Invitrogen), transferred to nitrocellulose membrane and stained with primary and secondary antibodies described above. Protein bands were visualized with Li-Cor Odyssey (Li-Cor Biosciences, Lincoln, NE).

### Tight junction barrier function and reassembly assays; Transepithelial Electrical Resistance, dextran flux and calcium-switch

Transepithelial Electrical Resistance (TER) and dextran flux was performed as previously described [30], however fluorescent-dextran concentrations in the well under the Transwell insert were quantified using SpectraMax M3 (Molecular Devices, Sunnyvale, CA) and experimental values was determined by extrapolation from the standard curve of known fluorescent-dextran concentrations using linear regression (GraphPad Prism, San Diego, CA). Calcium-switch was performed as described [28]. Statistical analysis (*t* tests or ANOVA as applicable) was performed using GraphPad Prism with corrections for multiple comparisons using the Sidak–Bonferroni method.

### Latrunculin B washout

SKco15 cells were plated at confluent density on collagen-coated Transwell filter inserts and grown to confluency overnight. Latrunculin B (Lat B; 10 μM), or DMSO as control, was added to both the top insert and the bottom well and after 2 hours of incubation cells were washed twice in complete culture media before adding fresh culture media for the indicated recovery times. Cells for the 0 minutes recovery were washed immediately in ice-cold PBS and fixed in 1%PFA in CSK buffer before immunostaining with rhodamine phalloidin and mouse anti-ZO-1 as described above. Cells were imaged with confocal microscopy at 20 X magnification.

### Wound-healing assay

Two techniques were used to measure wound-healing. First, we utilized the IncuCyte^®^ Zoom (Essen Bioscience, Ann Arbor, MI) to measure wound healing in a 96-well format. MDCKII cells (WT and SORBS2 KO) were plated at confluent density (35,000 cells/well) on a 96-well ImageLock plate (supplied by Essen Bioscience) the day before the experiment. The day of the experiment, a wound maker device (Essen Bioscience) was used to create a wound in each well of the 96-well plate. Cells were washed twice in complete media and either complete media or complete media containing 100 ng/μl HGF was added. Wound healing was tracked by imaging each well every 2h for up to 48h with a 10x objective. Essen Bioscience software was used to calculate the relative wound healing in each well. In the second method, we cultured SKco15 cells (WT and two different SORBS2 KO clones) on 60mm-dishes. A wound was created manually and cells were photographed at various timepoints and wound-healing was quantified in ImageJ as described previously [31].

## Results and discussion

### Defining SORBS2 isoforms expressed in human and canine epithelial cells

**Fig 1:**
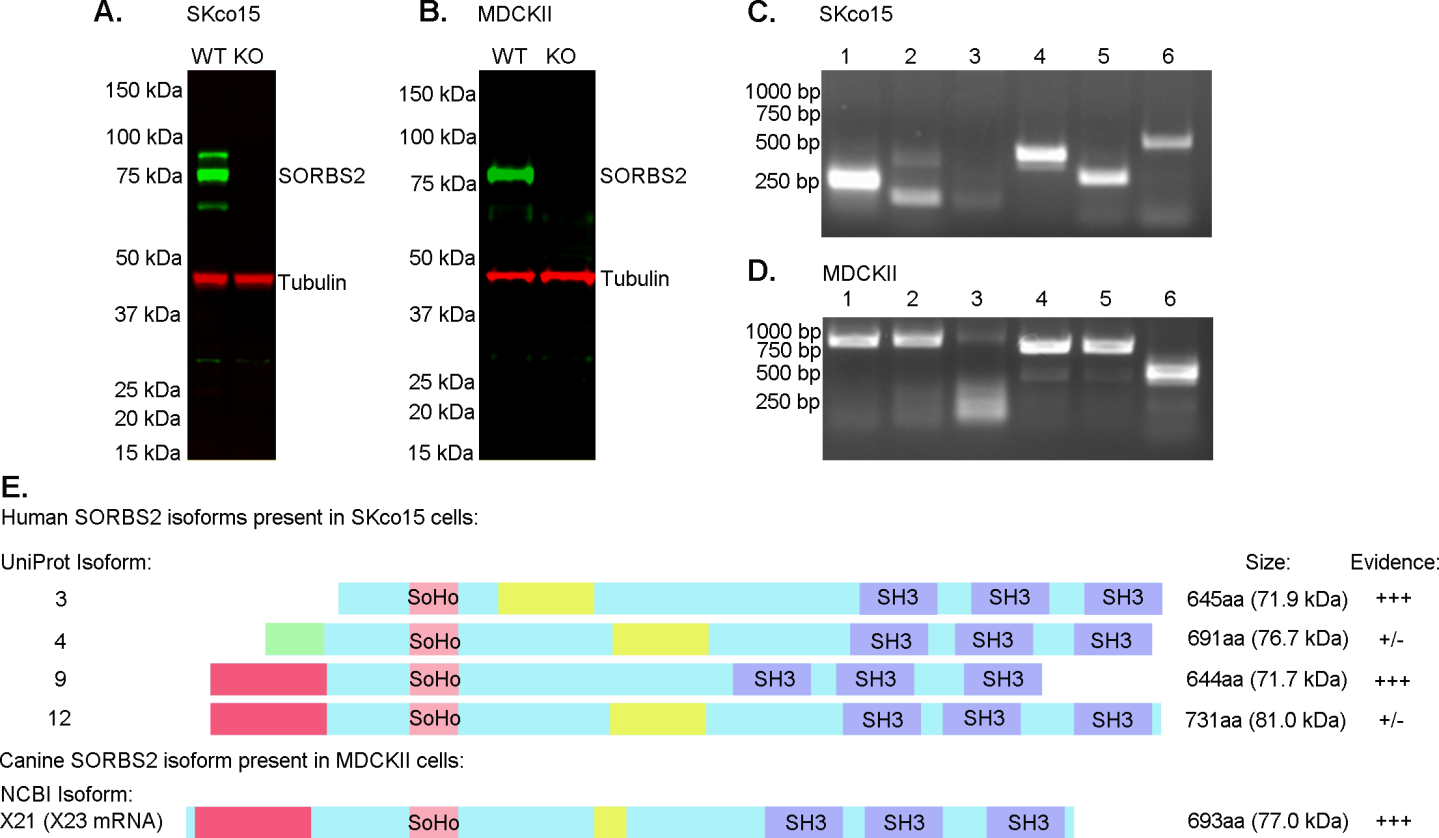
SORBS2 isoforms expressed in cultured human and canine epithelial cells. (A) Immunoblotted lysates from SKco15 cells reveal three distinct SORBS2 protein bands with sizes between approximately 65–85 kDa. After CRISPR KO SORBS2 protein is no longer detectable by immunoblot. (B) One distinct SORBS2 band, approximately 75 kDa in size, is visible in immunoblotted lysates from MDCKII cells and after CRISPR KO SORBS2 protein is no longer detectable. (C) DNA gel from PCR products generated by using six primer pairs to identify various isoforms of human SORBS2. Primer pair 1 is showing a 216 bp product, confirming isoform 9. Primer pair 2 is showing a distinct band at 185 bp consistent with isoform 9 and a weak band at 401 bp (could be isoforms 3, 4, 5, 12). Isoforms 2 and 8 can be excluded due to a lack of bands at 326 and 242 bp respectively. Isoform 2 can again be excluded due to the lack of a 176 bp product with primer pair 3. Primer pair 4 shows a 457 bp, confirming presence of isoforms 9 and 12. Primer pairs 5 and 6 both show the presence of isoform 3 at 303 bp and 540 bp respectively. (D) PCR products generated by using specific primers to identify various isoforms of canine SORBS2. Primer pair 1 should give a 900 bp product for isoform X23, which is confirmed. Isoform X25 would have been 878 bp, isoform X26 1109 bp and isoform X27 1177 bp respectively, which means these isoforms could be excluded by size. Isoform X25 has a very similar expected size to isoform X23, but we could exclude it based on the negative result obtained with primer pair 3. Primer pair 2 should give a 900 bp product for isoforms X23 and X26 which further confirms the presence of isoform X23. Primer pair 3 should identify isoforms X25 and X26 at 900 bp, but there is no evidence of a distinct product that size. We already excluded isoforms X25 and X27 and therefore the 850 bp product is thus isoform X23. Primer pairs 5 and 6 should recognize all potential SORBS2 isoforms (NCBI mRNA isoforms X23, X25, X26, X27) at 850 bp and 500 bp respectively. (E) Schematic figure of SORBS2 isoforms identified in human SKco15 cells and canine MDCKII cells. These are the only splice forms compatible with PCR results and the band sizes on the immunoblots.

We were unable to identify a reliable commercial antibody recognizing SORBS2 in epithelial cells, so we generated a custom rabbit anti-SORBS2 antibody to a conserved region between the first and second SH3 domains. This domain is present in all SORBS2 splice forms and thus an antibody directed against this region would be expected to identify all human splice forms, which range in size from 71–134 kDa (UniProt, [32]). The resulting antibody identified three distinct bands, e.g. at least three potential isoforms, of approximately 70, 75 and 80 kDa, in immunoblotted lysates from human intestinal epithelial SKco15 cells (Fig 1A) and one distinct band in canine kidney epithelial MDCKII cells around 75–80 kDa (Fig 1B). After utilizing the CRISPR-Cas9 system to knock out SORBS2 in SKco15 and MDCK cells, all of these bands were absent, consistent with their being authentic SORBS2 isoforms (Fig 1A (SKco15); Fig 1B (MDCKII)). As further evidence of antibody validation an immunofluorescence signal was observed at cell contacts and actin stress fibers in WT MDCKII cells, but there was no detectable signal in SORBS2 KO cells using the same confocal microscopy fluorescent sensitivity settings (S1 Fig, panel A).

Because our SORBS2 antibody recognized several different size bands in immunoblot analysis and both NCBI and UniProt [32] databases reported the existence multiple splice forms of SORBS2, we next attempted to identify the relevant splice forms expressed in MDCKII and SKco15 cells. Twelve human SORBS2 isoforms are reported in UniProt [32] and after eliminating isoforms 1, 6, 7 and 11 as being too large or too small to represent the observed bands we identified by immunoblotting, we designed six PCR primers pairs that could distinguish among mRNA transcripts encoding the remaining isoforms based on the size of the PCR products (primers and PCR product sizes are described in S2 Table). We confirmed the presence of mRNA for isoforms 3 and 9 in SKco15 cells based on the size of PCR products obtained from primer pair 5 and 6 (unique to isoform 3) and primer pair 2 (unique to isoform 9) and obtained weak signals for potential presence of mRNA encoding isoforms 4 and 12 with primer pair 2; (Fig 1C and 1E). Isoform 12 has the same expected size as isoform 9 with primer pair 4, which means it cannot be excluded. With the primers used, we did not observe PCR products of the size expected from isoforms 2, 5, 8 and 10 in SKco15 cells. In contrast to the many human isoforms predicted for human SORBS2, UniProt describes two canine SORBS2 isoforms predicted to be 134 kDa and 73 kDa, only the smaller of which might be expressed in MDCKII cells based on the size of the band detected by immunoblotting; several more isoforms are described in the NCBI database. By generating PCR primers to identify canine SORBS2 isoforms, in the observed size range between 70–85 kDa reported on the NCBI web site and UniProt, we confirmed expression of mRNA for isoform X23 (NCBI reference sequence XM_014120127.1), which corresponds to protein isoform X21 (NCBI reference sequence XP_ 013975602.1) in MDCKII cells (Fig 1D and 1E). Together, our results are most consistent with expression of splice forms 3, 4, 9 and 12 in SKco15 cells. MDCK cells express the canine mRNA isoform X23, which is most closely homologous to human isoforms 9 and 12. We note that all splice forms expressed in SKco15 and MDCKII epithelial cells contain the canonical SoHo domain and three SH3 domains; while other splice forms found in the databases are lacking various combinations of domains.

### SORBS2 is co-localized with apical junctional actin, and overlaps with both tight- and adherens junction proteins in polarized epithelial cells

**Fig 2:**
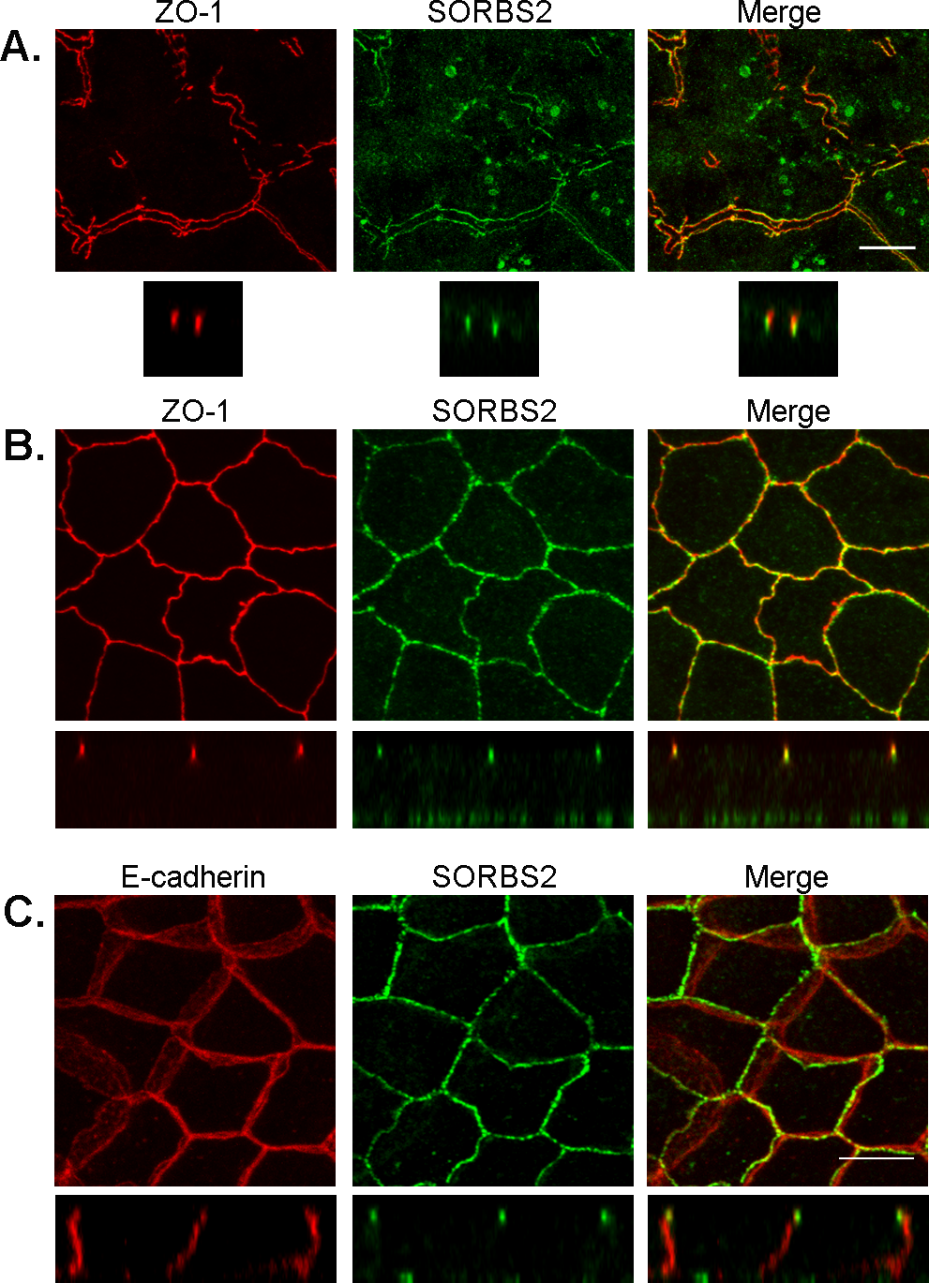
SORBS2 partially colocalizes with ZO-1 in murine bile canaliculi and with ZO-1 and E-cadherin in polarized MDCKII cells. (A) Confocal immunofluorescence analysis reveals that SORBS2 is localized to bile canalicular TJs in murine liver and is partially colocalized with ZO-1 in X, Y and Z planes (63x oil objective was used, scale bar: 10 μm). Images are maximum intensity projections of Z-stacks (total depth 4.5 μm). Merged images show that they overlap, but that ZO-1 also extends more apically. (B) SORBS2 is colocalized with ZO-1 and the apical portion of E-cadherin in polarized MDCKII cells cultured on Transwell filters for nine days (63x oil objective used, scale bar: 10 μm). Images are maximum intensity projections of Z-stacks (total depth for X-Y images ca 4.5 μm (to avoid the strong signal from basal actin stress fibers). SORBS2 is also faintly visible as green dots at the bottom of the cells (cross sections of actin stress fibers) in Z projections, especially in panel B. (full stack:10 μm).

The BioID results suggested that SORBS2 was proximal to the tight junction protein ZO-1 [7], as was consistent with localization previously reported in murine epithelial NMuMG cells [19]. We first verified the *in vivo* relevance of the BioID finding by confocal microscopy of SORBS2 in murine liver and found that SORBS2 and ZO-1 partially colocalize at TJs in bile canaliculi as shown in Fig 2A. This partial colocalization of SORBS2 and ZO-1 was also observed in MDCKII cells polarized on Transwell filters (Fig 2B) and SKco15 cells. Z-stacks revealed overlap between ZO-1 and SORBS2, but ZO-1 extends apically above, and SORBS2 basally below the region of overlap (Z-stack, Fig 2B). SORBS2 is also localized along basal actin stress fibers in MDCKII cells (Basal localization in Z-axis: Fig 2B, basal localization in X/Y axis: S1 Fig, panel B).

To ask if SORBS2 was also distributed at adherens junctions (AJ) we used E-cadherin (E-cad) as an AJ marker [33]. Even though SORBS2 was not found in BioID E-cad proteomic analysis [31,34] (S1 Table), we observed that SORBS2 indeed co-localizes with the most apical E-cad signal (Fig 2C). Our immunofluorescent confocal microscopy data thus indicates that SORBS2 overlaps with the basal region of the TJ and the apical region of the AJ in polarized MDCKII and SKco15 cells, similar to what has been described for the distribution of afadin [35].

**Fig 3:**
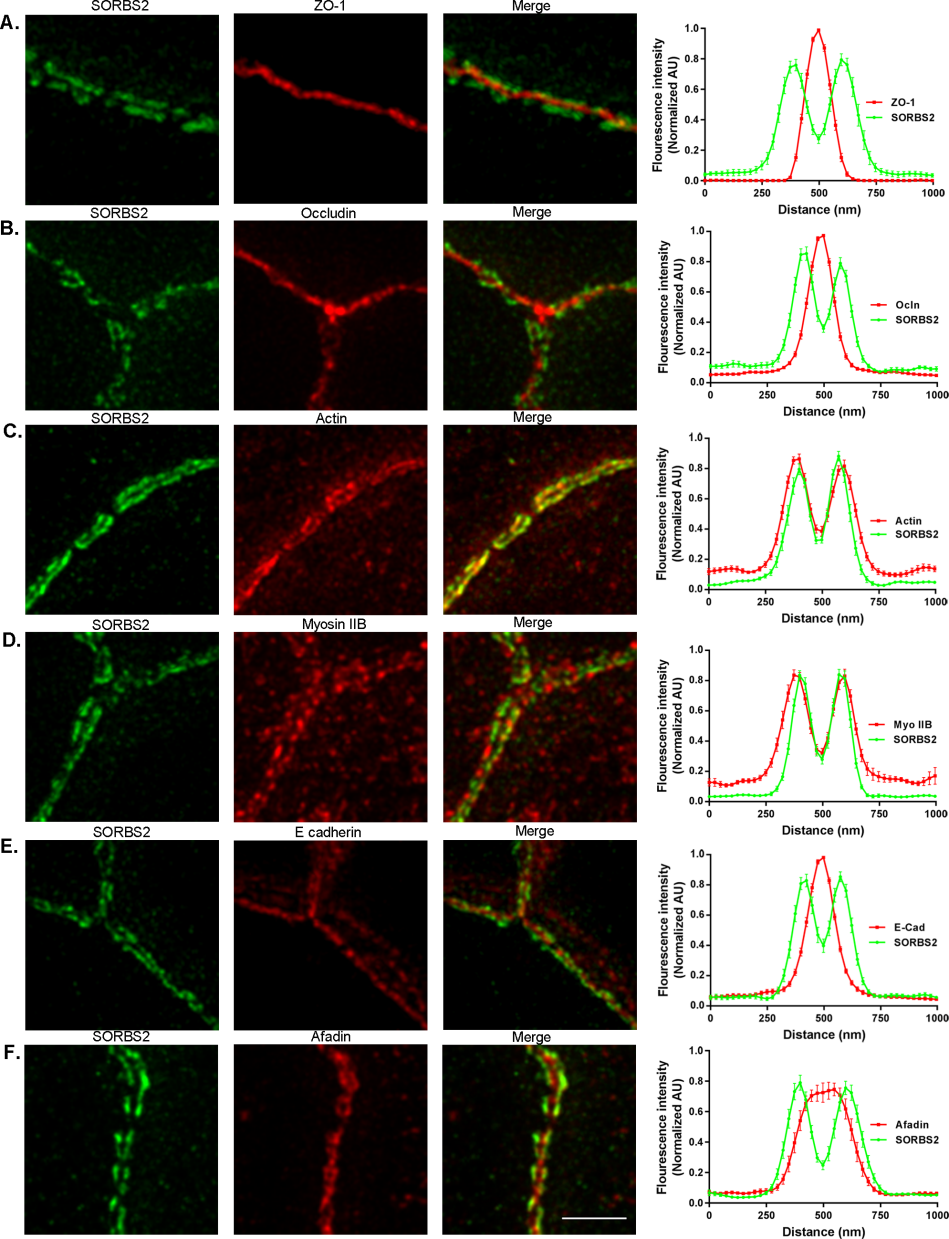
STED super resolution microscopy reveals that SORBS2 colocalizes with apical junctional actin and afadin, and partially overlaps with ZO-1, occludin and E-cadherin in polarized MDCKII cells. (A) Super resolution microscopy shows that SORBS2 in fact only partially colocalizes with ZO-1 in a discontinuous pattern along cell-cell contacts. Line scans of fluorescence intensity at 90 degrees to the cell contacts confirmed the visual observations, graph to right (N = 20, mean ± SEM). (B) As with ZO-1, SORBS2 is decorating occludin in a discontinuous pattern at cell-cell junctions and we noticed that SORBS2, at this level, is absent from tricellular junctions. (C) SORBS2 is colocalized with junctional actin, as confirmed both by visual appearance and line scan results. (D) Myosin IIB is localized in a discontinuous pattern at approximately the same distance from the cell-cell junction as SORBS2, but to note is that myosin IIB is present where SORBS2 is absent. (E) The very apical portion of E-cadherin is in the same X/Y plane as SORBS2, but SORBS2 localized farther from the membrane than E-cadherin. Scale bar: 0.5 μm. Images are maximum intensity projections of Z-stacks, total depth 2 μm.

To further define the spatial position of SORBS2 in relation to TJ, AJ and cytoskeletal proteins, we co-immunostained polarized MDCKII cells for actin, occludin, non-muscle myosin IIB, E-cadherin and afadin and performed STED super resolution microscopy. We observed that SORBS2 was localized more distal from the plasma membrane than ZO-1 and occludin, and unlike those proteins it has an irregular discontinuous pattern (Fig 3A and 3B). In order to approximate the distance of SORBS2 from the membrane relative to the other TJ proteins, we measured relative fluorescence intensity across the cell-cell junctions by line scanning at 90 degrees to the plane of cell contacts. The ZO-1 in opposing cells is sufficiently close that it cannot be visually resolved by super resolution methods; however, two peaks of SORBS2 signal are observed approximately 250 nm apart, each at about 125 nm inside of the cell-cell contact (Fig 3, right panels). SORBS2 also appears to be absent, or less abundant, at tricellular junctions (Fig 3B, 3D and 3E). SORBS2 was co-localized with the very apical actin at cell-cell contacts (Fig 3C), however actin is also distributed in small puncta over the apical cell surface and along the lateral membrane whereas SORBS2 immunofluorescence was confined to the perijunctional region (Fig 3C). It appears that myosin IIB, which also has a discontinuous pattern along the apical cell-cell contacts, is concentrated in the areas where SORBS2 is less abundant (Fig 3D), thus line scans had to be performed at different locations for SORBS2 and myosin IIB. Like actin, but unlike SORBS2, myosin IIB is also localized on the apical cell surface (Fig 3D). SORBS2 is also distributed distal from the membrane relative to the most apical E-cad signal (Fig 3E). The actin-binding protein afadin, which interacts with both ZO-1 and adherens junction components like nectin, partially colocalized with SORBS2 (Fig 3F). In contrast to myosin IIB, afadin and SORBS2 appear to co-concentrate in discontinuous junctional spots, although their co-localization is not complete (Fig 3F) [24].

The STED immunofluorescent analysis was designed for optimal x-y resolution, and we did not attempt Z-axis analysis, so the measurement of the x-y distributions are not meant to imply that these proteins are present in exactly the same apical-basal planes. In addition, the position of the antibody epitopes may not be centered on the protein and thus incompletely report the protein localization. With these caveats, SORBS2 appears to have a unique irregular discontinuous localization at the bicellular apical junction complex (TJ and AJ). SORBS2 is often co-localized with apical junctional actin and partially with afadin. SORBS2 is distributed overlapping with, but also partially basal to ZO-1 and occludin, but is lacking at tricellular junctions and co-distributes only with the most apical E-cad signal. This localization is distinct from the other TJ and AJ markers studied.

### SORBS2 is recruited to the sarcomere-like perijunctional actomyosin structure that is induced by knock-down of ZO-1 and ZO-2

**Fig 4:**
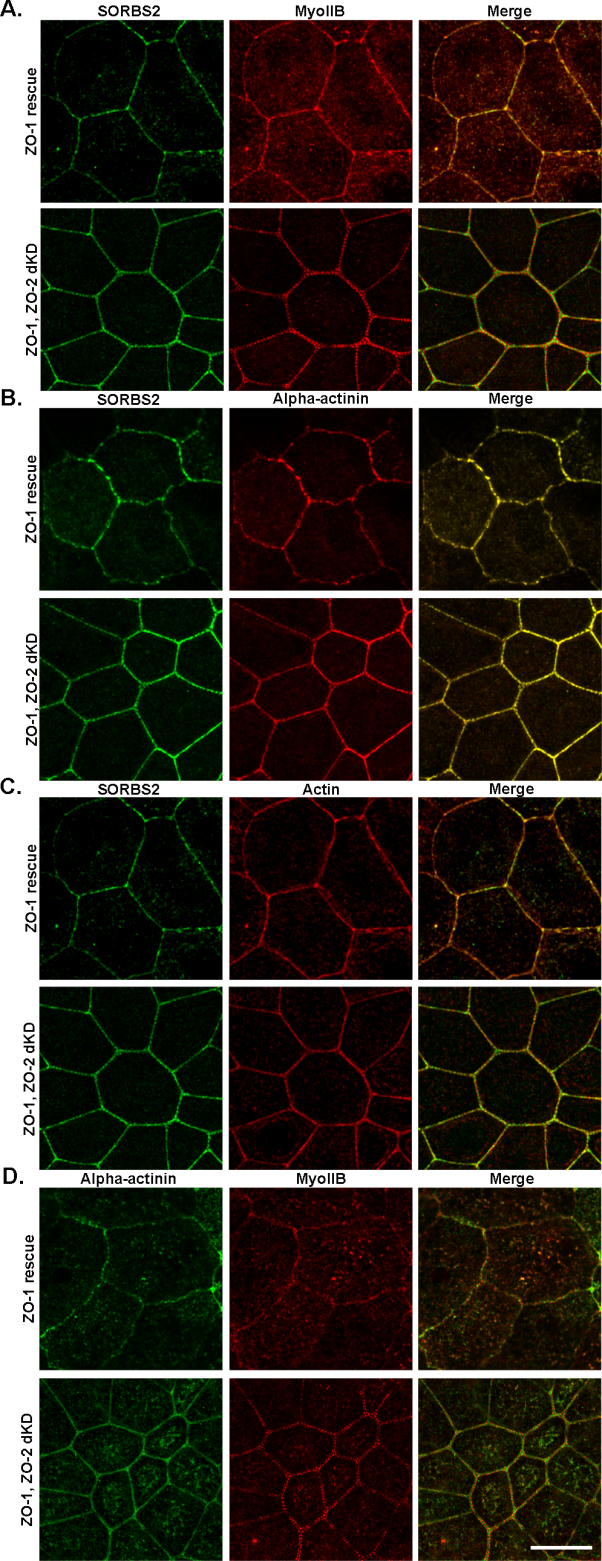
SORBS2 colocalizes with the thick contractile perijunctional actin, alpha-actinin and myosin structure induced by ZO-1, ZO-2 double knock-down in MDCKII cells. Super resolution Airyscan immunofluorescent images confirms that actin is redistributed in ZO-1, ZO-2 dKD cells (C, lower panel, middle image) and SORBS2 which is normally colocalized with apical junctional actin in MDCKII cells (C, upper panel) is now redistributed with actin (C, lower panel, left and right images). Myosin IIB and alpha-actinin are also redistributed in ZO-1, ZO-2 dKD cells (Myosin IIB: A, lower panel center and D, lower panel center; alpha-actinin: B, lower panel center and D, lower panel left) as compared to cells rescued with ZO-1 (Myosin IIB: A and D, top panel center; alpha-actinin: B and D, top panel center). SORBS2 appears to co-localize with alpha-actinin and mostly colocalize with actin in both ZO-1 rescue cells and ZO-1, ZO-2 dKD cells (alpha-actinin: B, right images in upper and lower panel; actin: C, right images in upper and lower panel). Myosin IIB in ZO-1, ZO-2 dKD cells decorate the distal part of both SORBS2 (A: right image in lower panel) and alpha-actinin (D: right image in lower panel). (63x oil objective used, scale bar: 20 μm). Images are maximum intensity projection of Z-stacks (total depth range: 2–3.3 μm).

The evidence above indicates that SORBS2 is a structural component of the perijunctional acto-myosin ring. We previously showed that double knock-down (dKD) of ZO-1 and ZO-2 in MDCKII cells leads to a striking sarcomere-like organization of non-muscle myosin II which is associated with increased tension at the AJ [26]. Because SORBS2 is a known component of Z-bands in cardiomyocytes [10,13], we asked whether SORBS2 co-accumulates with the contracted sarcomere-like structure. Like several other actin-binding proteins [26], SORBS2 becomes concentrated in the expanded perijunctional cytoskeletal array in the ZO-1, ZO-2 dKD cells (Fig 4A, 4B and 4C, bottom panels). Its distribution is similar to alpha-actinin (Fig 4B, bottom right) and similar, but not precisely identical, with actin (Fig 4C, bottom right). Myosin IIB alternates with SORBS2 and alpha-actinin but lies relatively distal to both these proteins (Fig 4A and 4D, bottom right panels respectively). The significance of the non-overlapping distribution of SORBS2 and myosin IIB is unclear, but one possibility is that the reported interaction of SORBS2 with alpha-actinin [21,36], which has a similar non-overlapping relationship to myosin IIB in sarcomeric acto-myosin arrays in some epithelial cells [37], may be guiding this organization. This is also in agreement with previous findings in non-epithelial cells [38–42] showing that the domains occupied by myosin filaments were separated by zones enriched in the actin crosslinking protein alpha-actinin (isoforms 1 and 4). However, we found that in the ZO-1, ZO-2 dKD cells, the immunofluorescent signal for myosin IIB is more displaced from the membrane compared with wild-type cells (~400 nm spread on either side of the membrane compared with ~ 250 nm in wild-type MDCKII cells), while there is less detectable change in the distribution of SORBS2 or α-actinin. This suggests that the observed arrangement of SORBS2 does not fully recapitulate sarcomeric organization. In ZO-1, ZO-2 dKD cells rescued by inducible expression of a ZO-1 transgene, actin, myosin IIB, alpha-actinin and SORBS2 localizations are normalized (Fig 4A, 4B, 4C and 4D, top panels) and resemble wild-type localization at cell-cell junctions. Together these results suggested that SORBS2 might be responding to changes in epithelial tension caused by the ZO-1, ZO-2 dKD [43].

**Fig 5:**
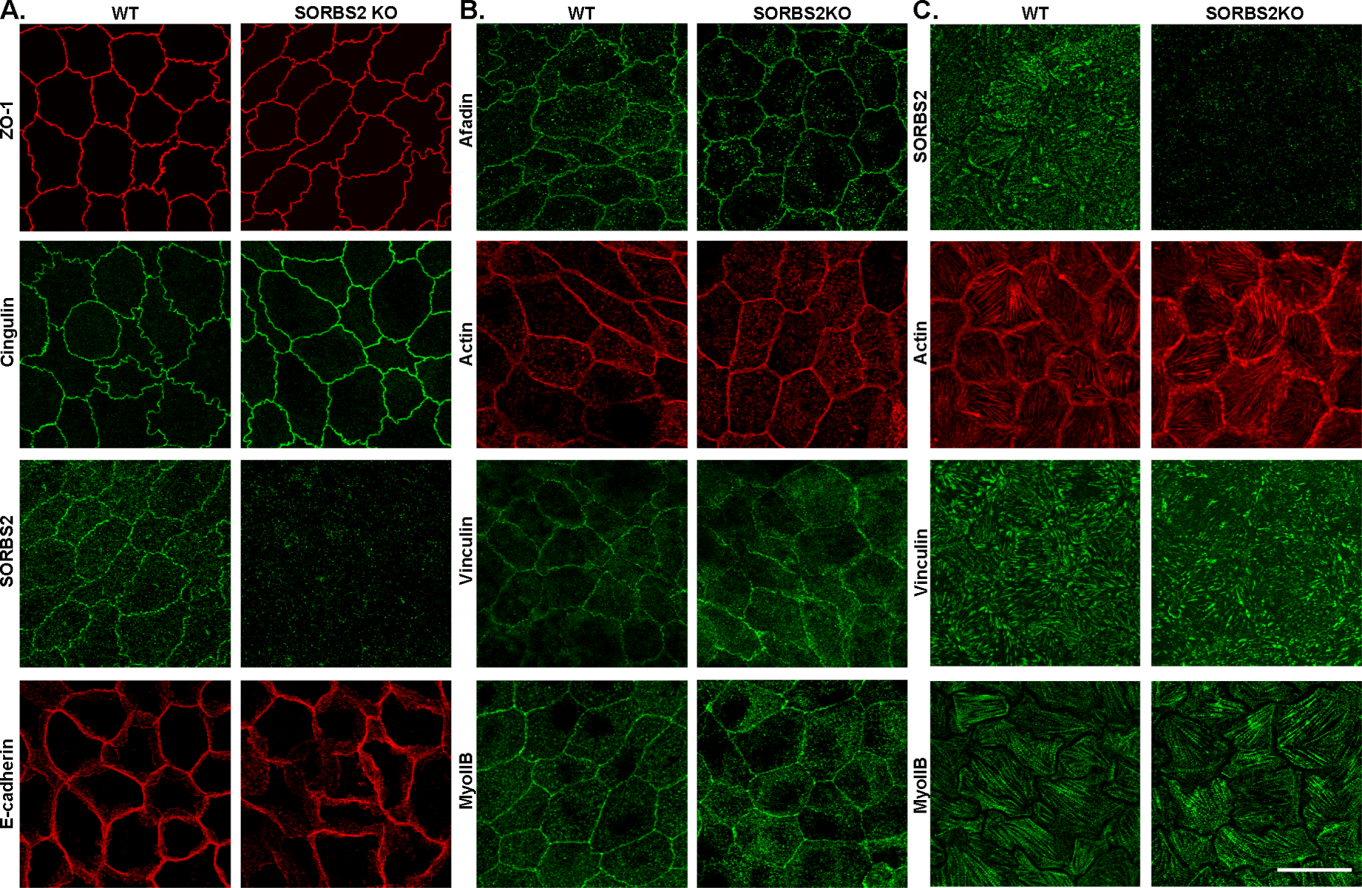
Tight junction, adherens junction and cytoskeletal proteins are not changed either at cell-cell contacts or at actin stress fibers in SORBS2 knock-out cells. (A) Localization of the TJ proteins ZO-1 and cingulin is not changed in SORBS2 KO cells (right panel) as compared to WT cells (left panel). SORBS2 is present in a discontinuous pattern at apical cell-cell contacts in WT cells (left panel) and is not visible in SORBS2 KO cells (right panel). The adherens junction proteins E-cadherin (panel A) and afadin (panel B) are both unchanged by SORBS2 KO as compared to WT cells (right and left panels respectively). (B) the cytoskeletal proteins actin, vinculin and myosin IIB are also not affected by SORBS2 KO (right panel) as compared to WT cells (left panel). (C) SORBS2 is also localized at actin stress fibers (WT, left panel) and is not visible in SORBS2 KO cells (right panel). Actin, vinculin and myosin IIB are not changed at actin stress fibers in SORBS2 KO cells (right panel) as compared to WT cells (left panel). 63x objective was used, apical images are maximum intensity projections (depth range: 1.7–3 μm depending on the basolateral distribution of each protein, scale bar: 20 μm). Basal images: SORBS2 and actin are maximum intensity projections (1.26 μm), vinculin (0.82 μm) and Myosin IIB (0.42 μm). Scale bar: 20 μm.

We previously showed that knock-out of the ZO-1 binding protein TOCA-1 also results in a striking accumulation of contractile actin and myosin at the AJ [28]. To test if, like TOCA-1, SORBS2 regulated the perijunctional actomyosin cytoskeleton, we generated SORBS2 knockout cell lines (Fig 5A, 5B and 5C; right panels) and compared the apical junctional organization of actin, myosin IIB, vinculin, afadin, E-cadherin, cingulin and ZO-1 as well as actin, myosin IIB and vinculin at actin stress fibers in WT and SORBS2 KO MDCKII cells. Distribution of these proteins was unaffected by deleting SORBS2 (apical distribution: Fig 5A and 5B; actin stress fiber distribution: Fig 5C). These results indicate that although SORBS2 is known to bind to actin, alpha-actinin and vinculin, it is not required to create or maintain the normal location of these proteins at apical cellular junctions or actin stress fibers.

SORBS2 has been reported to interact with alpha-actinin [36,44], which has been implicated in the regulation of actin assembly at cell junctions [45,46]. To determine if SORBS2 interaction with actin and/or alpha-actinin might be implicated in apical junctional stabilization or reassembly, we used Latrunculin B (Lat B) treatment to depolymerize actin filaments and visualized the recovery the apical junction organization indirectly by measuring ZO-1 immunofluorescent staining after 0, 60, 120 and 240 minutes following Lat B removal in WT and SORBS2 KO SKco15 cells. This protocol has previously been used to demonstrated a requirement for α-actinin-4 in apical junctional reassembly [45]. Lat B treatment in both WT and SORBS2 KO cells resulted in the previously reported fragmented pattern of junctional ZO-1 [47,48] (S2 Fig, panel B). Junctional recovery, assessed by ZO-1 immunofluorescent staining was not significantly different between WT and SORBS2 KO cells (S2 Fig, panel C), suggesting that SORBS2 is not a critical component of actin stability or reassembly at cell-cell contacts.

### Knock-out of SORBS2 does not significantly change epithelial tight junction barrier function- or reassembly as measured by transepithelial resistance, dextran flux and calcium-switch in epithelial cells

Because SORBS2 was identified as proximal to both ZO-1 and occludin and co-localized with these proteins at tight junctions, we tested to see if we could detect a physiologic role of SORBS2 on tight junction barrier function. We found no difference in either transepithelial resistance or flux of a 3kDa fluorescent dextran between WT and SORBS2 KO in either MDCKII or SKco15 cell backgrounds. The data do not support a requirement for SORBS2 in regulation of normal baseline barrier function.

**Fig 6:**
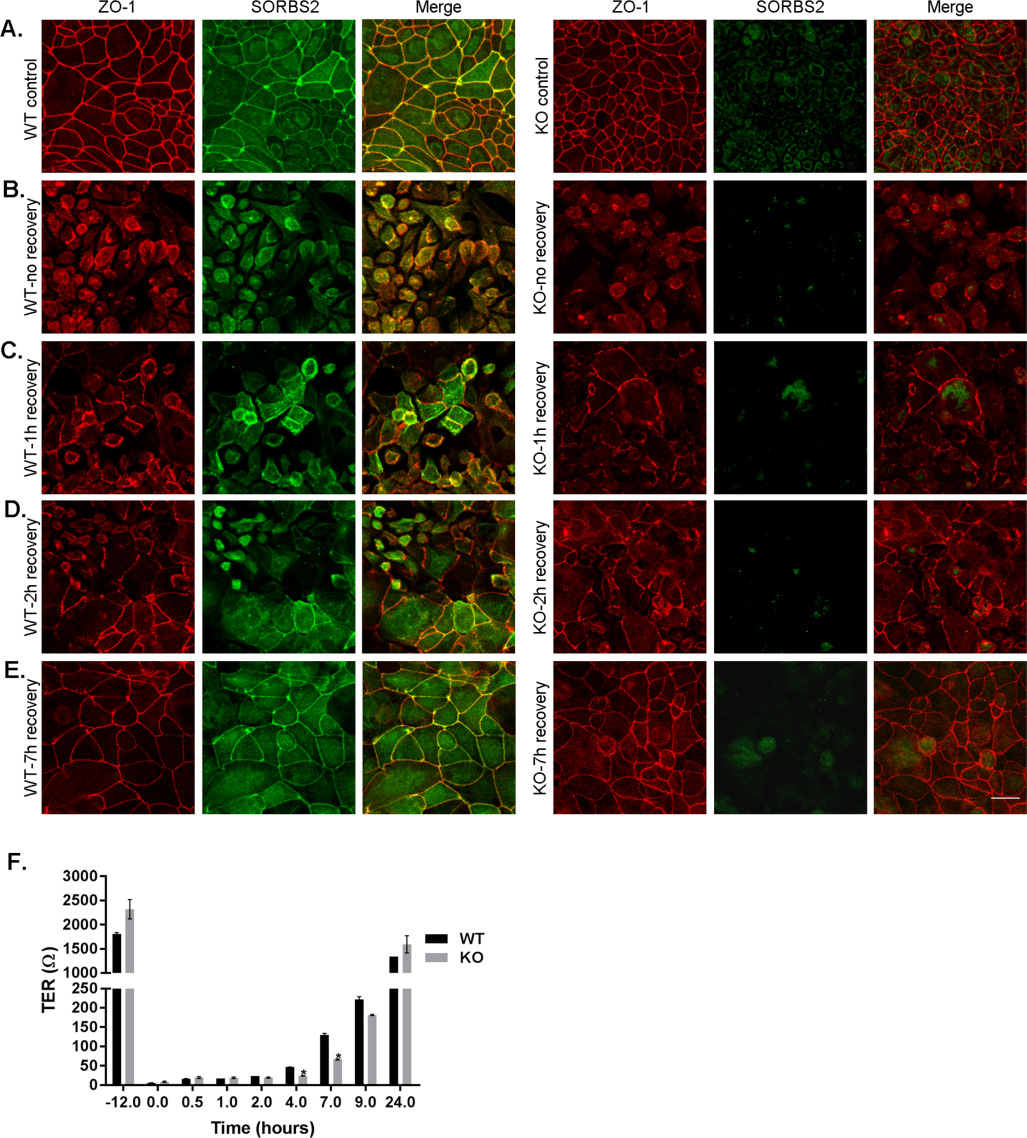
Knock-out of SORBS2 does not affect TJ recovery after calcium switch. (A) SORBS2 colocalizes with ZO-1 in WT SKco15 cells under normal culture conditions. (B) After overnight culture in low-calcium both WT and SORBS2 KO cells are rounded up and no normal cell-cell junctions are visible. Already one hour after normal calcium is restored cell-cell junctions are starting to recover (C) and the recovery progresses after 2 hours (D) and after 7 hours (E) the cell-cell contacts look normal by immunofluorescent microscopy. Scale bar: 20 μm. Images are maximum intensity projections of Z-stacks (total depth:10 μm). (F) TER is high in both WT and SORBS2 KO SKco15cells before calcium removal. However, after overnight low calcium TER is almost undetectable. TER recovers as the cell-cell contacts are reassembled and is almost fully recovered 24 hours after normal calcium is restored. Data shown are mean ±SEM of duplicate samples.

Removal of calcium from cell culture media disrupts cell-cell contacts that are reassembled after normal calcium concentration is restored [26,49–51]. Cell-cell junction assembly and reassembly has been shown to be dependent on actin [52,53] and a number of actin interacting proteins [51]. We performed a calcium switch assay to assess whether SORBS2 is involved in reassembly of the apical cell-cell contacts when normal calcium is restored after culture in low calcium. As expected, immunofluorescence images of WT and SORBS2 KO SKco15 cells show TJ disassembly after overnight incubation in low calcium media (Fig 6B, WT left and SORBS2 KO right panels) as compared to control in normal calcium (Fig 6A). One hour after normal calcium concentrations was restored, ZO-1 is present, although discontinuous, at cell-cell contacts both in WT and SORBS2 KO cells (Fig 6C). SORBS2 in WT cells co-localizes with ZO-1 at the reforming TJs (Fig 6C, left panel). Both ZO-1 and SORBS2 immunofluorescent signal continues to elongate at cell-cell contacts and by 7h the whole epithelial layer is visually intact with continuous junctions (Fig 6D and 6E). Recovery of transepithelial resistance after calcium-switch was not significantly different in SORBS2 KO SKco15 cells as compared to WT control cells (Fig 6F). Taken together, these results do not suggest a requirement for SORBS2 in reassembly of the tight junction barrier and restoration of its function.

### SORBS2 is not required to mediate PDGF-induced paracellular flux in epithelial cells

One reported role for SORBS2 is as a scaffolding protein in pathways associated with Abl/Arg non-receptor tyrosine kinases. Platelet Derived Growth Factor (PDGF) activates c-Abl which then can phosphorylate WAVE proteins and regulate their activity. Phosphorylation of WAVE induced by overexpression of c-Abl has been shown in pancreatic cultured cells to be transduced by the presence of SORBS2 [25]; the interpretation was that SORBS2 can scaffold these proteins and thus facilitate their mutual interaction. PDGF has been shown to increase permeability in MDCKII cells [54] and therefore we hypothesized that SORBS2 might be required to mediate permeability changes in response to PDGF treatment. However, both WT and SORBS2 KO cells responded equivalently with increased flux of 3 kDa dextran after PDGF-BB treatment (baseline flux WT: 0.11 ± 0.02, SORBS2 KO 0.16 ± 0.03 ug/ml; post PDGF treatment, WT: 0.76 ± 0.05, SORBS2 KO 1.07 ± 0.21 μg/ml, (mean ± SEM from triplicates)). A similar lack of effect was seen when flux was measured with 70kDa dextran (baseline flux 70 kDa dextran: WT: 0.05 ± 0.03, SORBS2 KO 0.05 ± 0.04 baseline flux; WT: 0.18 ± 0.02, SORBS2 KO 0.18 ± 0.02 ug/ml in response to PDGF-BB, (mean ± SEM from triplicates)). The results failed to support the requirement for SORBS2 in mediating PDGF-induced changes in barrier function.

### Knock-out of SORBS2 does not change HGF-induced cell scattering or wound healing properties

**Fig 7:**
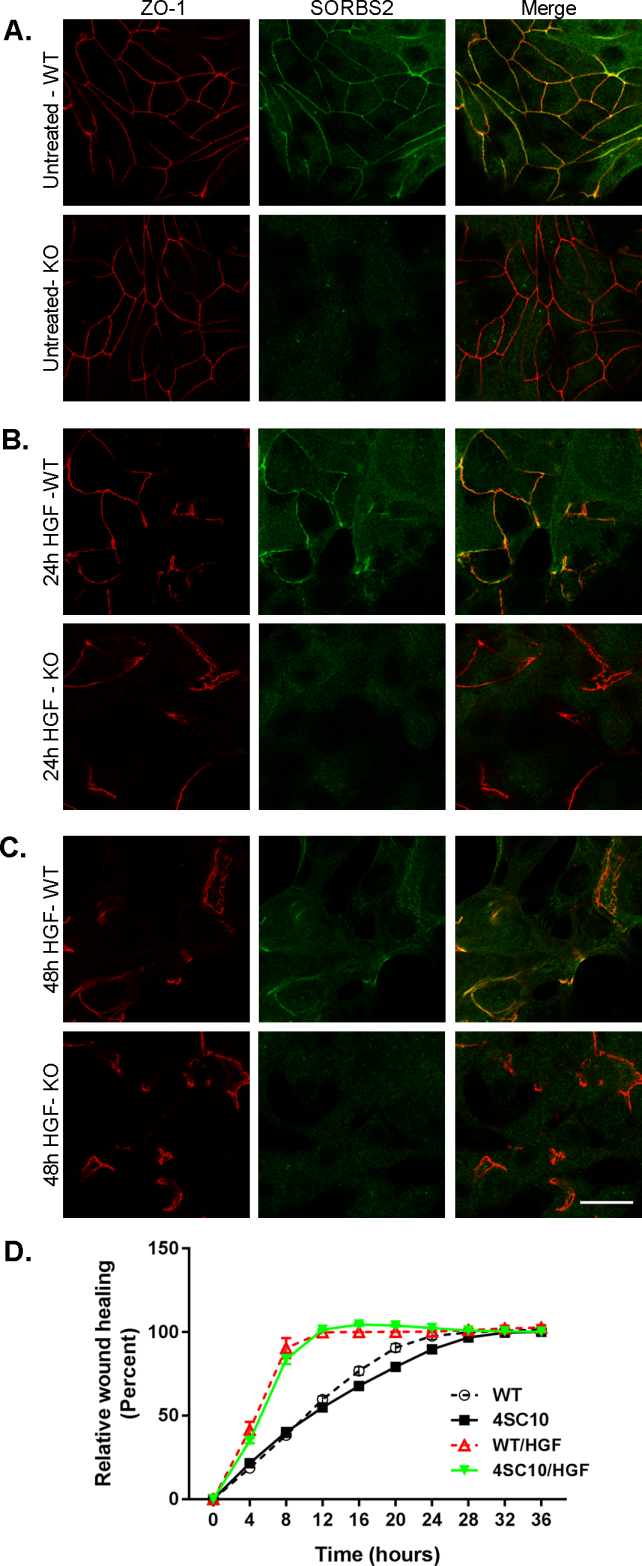
HGF-induced cell scattering and accelerated wound healing is the same in SORBS2 knock-out and control MDCKII cells. (A) SORBS2 colocalizes with ZO-1 in WT MDCKII cells. (B) Twenty-four hours after HGF treatment cells scatter and ZO-1 becomes discontinuous at cell-cell contacts in both WT and SORBS2 KO cells, as does SORBS2 in WT cells. (C) Forty-eight hours after HGF treatment cell scattering patterns again looks the same in both WT and SORBS2 KO cells. (D) HGF speeds up wound healing in both SORBS2 KO and WT cells (12 hours) as compared to normal complete media (28 hours), but there is no difference between SORBS2 KO and WT MDCKII cells (63 x oil objective, scale bar: 20 μm).

Abl/Arg non-receptor tyrosine kinases are activated downstream of the Met RTK receptor, which is the receptor for hepatocyte growth factor (HGF) [55]. Inhibition of Abl kinases has been shown to suppress HGF-induced cell scattering, tubulogenisis and migration [55]. Since SORBS2 binds Abl/Arg [10], and is also a substrate for these tyrosine kinases [18], we hypothesized that knocking out SORBS2 would potentially reduce HGF-induced cell scattering or the rate of wound healing in MDCKII cells. After adding HGF, and incubating cells for 24 and 48 hours, we observed no difference in the degree of HGF-induced cell scattering in WT cells (Fig 7A, 7B and 7C, top panels) compared with SORBS2 KO cells (Fig 7A, 7B and 7C, bottom panels) visualized by immunostaining of ZO-1 and SORBS2. We further confirmed this observation with phase images of WT and SORBS2 KO SKco15 cells. A wound healing assay, with and without HGF added, revealed no significant difference in the ability of SORBS2 KO MDCKII cells to close the wound as compared to WT control cells (Fig 7D), although adding HGF significantly sped up the wound closure for both WT and KO cells (12 hours with HGF present and 24h under normal culture conditions for both WT and SORBS2 KO respectively).

Our data shows that even though SORBS2 is reported to be a target for Abl/Arg [18,36], knocking it out does not alter cell scattering or wound healing after HGF stimulation in MDCKII cells.

### SORBS1 and SORBS3 expression is not increased in SORBS2 knock-out epithelial cells

It has been previously demonstrated that SORBS1 KO cells show compensatory increases in SORBS2 expression [56]. We found by BioID that SORBS1 was identified as proximal to both the N- and C-terminal of ZO-1 and occludin (number 91 and 98 respectively, compared to 16 for SORBS2 at the N-terminus of ZO-1), at the C-terminus of occludin (number 344) (S1 Table) [8,9,31]. One possibility was that an increase in SORBS1 in SORBS2 KO cells could compensate for the loss of SORBS2, resulting in all our negative findings. However, immunoblotted lysates from both MDCKII and SKco15 cells showed barely detectable and equivalent levels of SORBS1 both in WT and SORBS2 KO cells, indicating that SORBS1 was not increased in SORBS2 KO cells. Antibody specificity was verified by transfecting HEK293 Tet-Off^®^ Advanced cells with myc-DDK-SORBS1 (S3 Fig). Low basal levels and lack of a compensatory induction was further verified by qRT-PCR of SORBS1 mRNA in MDCKII cells (WT: 0.03 (mean of 2^-delta Ct normalized to ZO-1) vs. SORBS1 mRNA in SORBS2 KO cells: 0.02)). In comparison SORBS2 mRNA in WT MDCKII cells was almost 20-fold higher at 0.54 (2^-delta Ct normalized to ZO-1). In addition, although we (S1 Table) and others [34] failed to identify SORBS3 as a tight or adherens junction protein by proteomic analyses, to exclude the possibility that SORBS3 could compensate for the loss of SORBS2, we performed qRT-PCR in WT and SORBS2 KO MDCKII cells. Our results show that SORBS3 was not increased in the SORBS2 KO MDCKII cells ((1.04) (2^-delta Ct normalized to ZO-1) as compared to WT TO-MDCKII cells (1.12).

Since neither SORBS1 nor SORBS3 expression increases in SORBS2 KO cells, it seems unlikely that they compensate for the deletion of SORBS2.

### Transiently expressed GFP-SORBS2 recruits actin, alpha-actinin, vinculin and N-WASP in MDCKII cells

**Fig 8:**
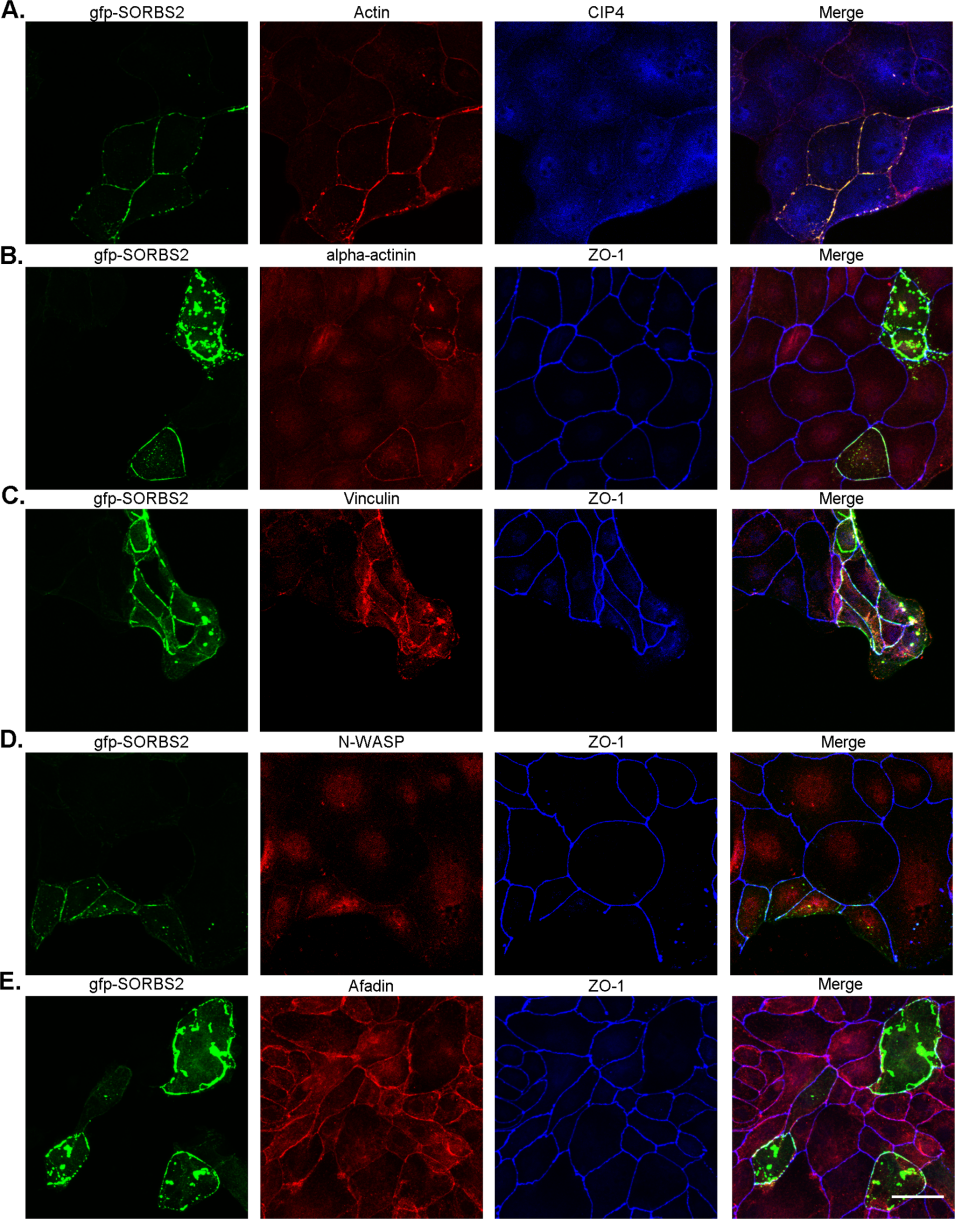
GFP-SORBS2 expression in MDCKII cells recruits actin, alpha-actinin, vinculin, N-WASP and possibly CIP4. Overexpression of GFP-SORBS2 is strongly associated with accumulation of actin, alpha-actinin and vinculin (A, B, C) and weakly associated with N-WASP and possibly CIP4 (D, A) as shown by confocal immunofluorescence imaging. Afadin accumulation was not induced by expression of gGFP-SORBS2 (E). 20x objective was used, scale bar: 40 μm. Images shown are maximum intensity projection of Z-stacks (total depth: 10 μm).

SORBS2 has previously been shown to not only interact with actin [10,13,14,18,19,21,36], but also with a variety of junctional actin-binding proteins, including alpha-actinin [13,21,36], vinculin [13,18,21,24], N-WASP [19], CIP4 [17] and afadin [24]. We first attempted co-immunoprecipitations (co-IP) to verify reported interacting partners of SORBS2, but we were unable to detect previously described interaction partners using several co-IP different protocols. We therefore used an alternative functional approach and asked whether transient over-expression of SORBS2 in MDCKII cells resulted in the recruitment of actin and junctional actin binding proteins. We transfected both WT and SORBS2 KO MDCKII cells with GFP-SORBS2 and 72h post transfection the cells were fixed and stained for actin, alpha-actinin, vinculin, N-WASP, afadin, CIP4 and ZO-1. In cells expressing GFP-SORBS2 there was a clear recruitment of additional actin, alpha-actinin and vinculin in both WT cells (Fig 8A, 8B and 8C) and SORBS2 KO cells (S4A, S4B and S4C Fig) and weak recruitment of junctional N-WASP (Fig 8D, S4D Fig) and CIP4 (Fig 8A, S4A Fig). To our surprise there was no concentration of afadin with over-expressed SORBS2, previously reported as a SORBS2 interacting protein (Fig 8E, S4E Fig). ZO-1 localization was unaffected by both SORBS2 KO and over-expression of GFP-SORBS2 (Fig 8B, 8C, 8D and 8E; S4B, S4C, S4D and S4E Fig). SORBS2-dependent recruitment of these proteins in over-expressing cells may be due to direct binding of these proteins to SORBS2 or to an indirect interaction, for example concentration secondary to actin association.

Perijunctional actin is critical for maintaining the tight junction barrier and in this study, we attempted to define a role for the actin-binding junctional protein SORBS2 in regulating the structure and function of this barrier. This interest was based on our previous BioID finding that SORBS2 is close to ZO-1, and on several publications supporting a role for SORBS2 as a scaffolding protein in tyrosine kinase pathways that control the actin cytoskeleton and cell motility. We were able to show that SORBS2 indeed is located in the contractile perijunctional actomyosin ring and overlaps both the tight junction and adherens junction, and that in contrast to the continuous distribution of other junction proteins; it shows a peculiar irregular discontinuous distribution. However, deletion of SORBS2 in two different epithelial cell lines had no effect, relative to control cells, on multiple standard assays of tight junction barrier property, actin assembly and HGF or PDGF effects on cell motility, cell spreading, or barrier function. At this point the role of SORBS2 in the apical junction complex remains undefined.

## Supporting information

S1 Fig SORBS2 antibody specificity. Immunofluorescent staining with our custom rabbit-anti SORBS2 antibody showed apical SORBS2 staining in WT MDCKII cells ((A), top right and top left panels) and basal staining ((B, top panel). ZO-1 was used as an apical TJ maker ((A), middle and right panels). SORBS2 immunofluorescence was not detectable either apically at TJ ((A), bottom left and right panels) or basally ((B), bottom panel)) in SORBS2 KO MDCKII. Scale bar: 10 μm.
(DOCX)

S2 Fig ZO-1 recovery is not changed after Latrunculin B washout in SORBS2 knock-out cells. Untreated control cells (WT and SORBS2 KO SKco15) or cells exposed to 10uM Latrunculin B for 2 hours, followed by washout and recovery for 30 or 60 minutes, were immunofluorescently labeled with a ZO-1 antibody. Confocal imaging reveled normal ZO-1 localization in control cells (panel A), disrupted ZO-1 localization 30 minutes after Latrunculin B washout (panel B) and partially recovered ZO-1 staining after 60 minutes (panel C). However, there was no difference in recovery between WT and SORBS2 KO cells. 20x objective was used, images are maximum intensity projections (depth: 8.8 μm), scale bar: 20 μm.
(DOCX)

S3 Fig SORBS1 antibody verification. To verify antigen recognition and specificity of the SORBS1 antibody we transfected HEK293 cells with myc-DDK-tagged human SORBS1 and immunoblotted the cell lysate in parallel with wild type cell lysate. The SORBS1 antibody used did indeed recognize the SORBS1 fusion protein as well as an endogenous smaller band around 70–75 kDa. There are 12 isoforms of human SORBS1 listed in the Uniprot database ranging in size between 68.7–143 kDa. Two of the 12 isoforms are close to the size identified in wild type HEK293 cells (isoform 4: 76.6 kDa and isoform 7: 68.7 kDa).
(DOCX)

S4 Fig GFP-SORBS2 overexpression in SORBS2 knock-out MDCKII cells show accumulation of actin, alpha-actinin, vinculin, N-WASP and possibly CIP4. As in WT MDCKII cells, expression of GFP-SORBS2 in SORBS2 KO cells is strongly associated with accumulation of actin, alpha-actinin and vinculin (A, B, C) and weakly associated with N-WSAP (D) and possibly CIP4 (A) as shown by confocal immunofluorescence. Afadin accumulation was not associated with GFP-SORBS2 (E). Scale bar: 40 μm.
(DOCX)

S1 Table Summary of our previously published BioID tagging results showing the enrichment of SORBS2 around tight- and adherens junction proteins. Numbers shown are rank order of the frequency of tagging based on averages of three independent experiments, calculated by normalized peptide-spectrum match divided by observable peptide number as described [8, 9, 30]. ND = not detected. For example, ZO-1 is the #1 protein tagged by biotin ligase fused to the N-terminus of ZO-1 and SORBS2 is #16, higher than the well described TJ protein claudin-4 at position #25. Of the BioID constructs tested SORBS2 is most enriched at the N-terminus of ZO-1. All rank numbers in this table are based on enriched proteins, e.g. we removed all proteins that were three times or less above the biotin ligase alone levels. See references for details [8, 9, 30].
(DOCX)

S2 Table SORBS2 sgRNA for CRISPR Cas9 knock-out, SORBS2 sequencing primers, SORBS2 PCR primers for isoform identification, qRT-PCR primers for SORBS1, SORBS2 and SORBS3 and InFusion primers for inserting SORBS2 in the EGFP C1 vector. (DOCX)
