# Supplementary figures and images for: Sorbin and SH3 domain-containing protein 2 (SORBS2) is a component of the acto-myosin ring at the apical junctional complex in epithelial cells
Source: PLoS One. 2017 Sep 29;12(9):e0185448. doi: 10.1371/journal.pone.0185448 (PMC5621683; doi:10.1371/journal.pone.0185448)

**S1 Fig.**

**
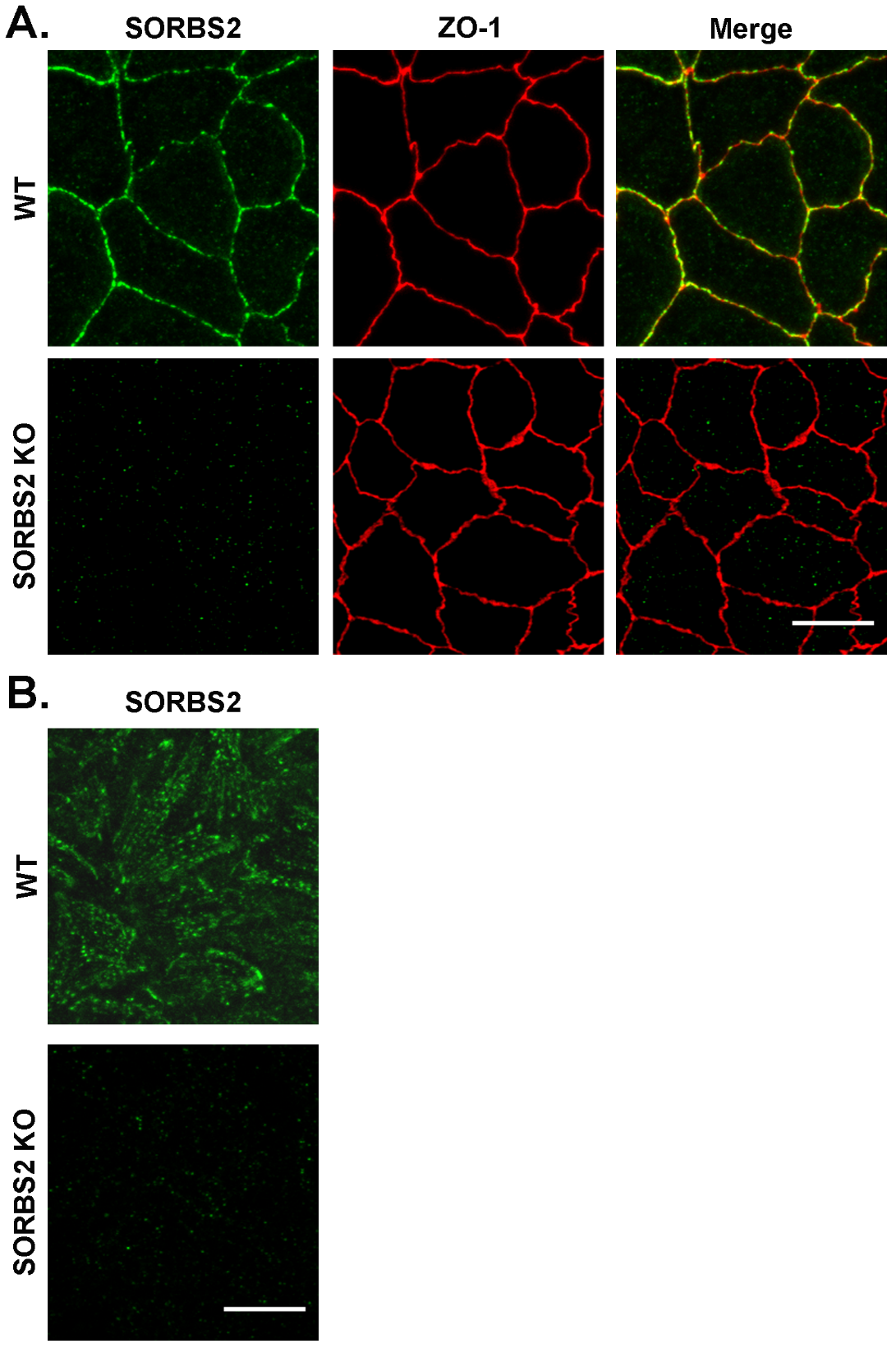
**

Supplement: S1 Fig — Immunofluorescent staining with our custom rabbit-anti SORBS2 antibody showed apical SORBS2 staining in WT MDCKII cells ((A), top right and top left panels) and basal staining ((B, top panel). ZO-1 was used as an apical TJ maker ((A), middle and right panels). SORBS2 immunofluorescence was not detectable either apically at TJ ((A), bottom left and right panels) or basally ((B), bottom panel)) in SORBS2 KO MDCKII. Scale bar: 10 μm. (DOCX) [file pone.0185448.s001.docx]

**S2 Fig.**

**
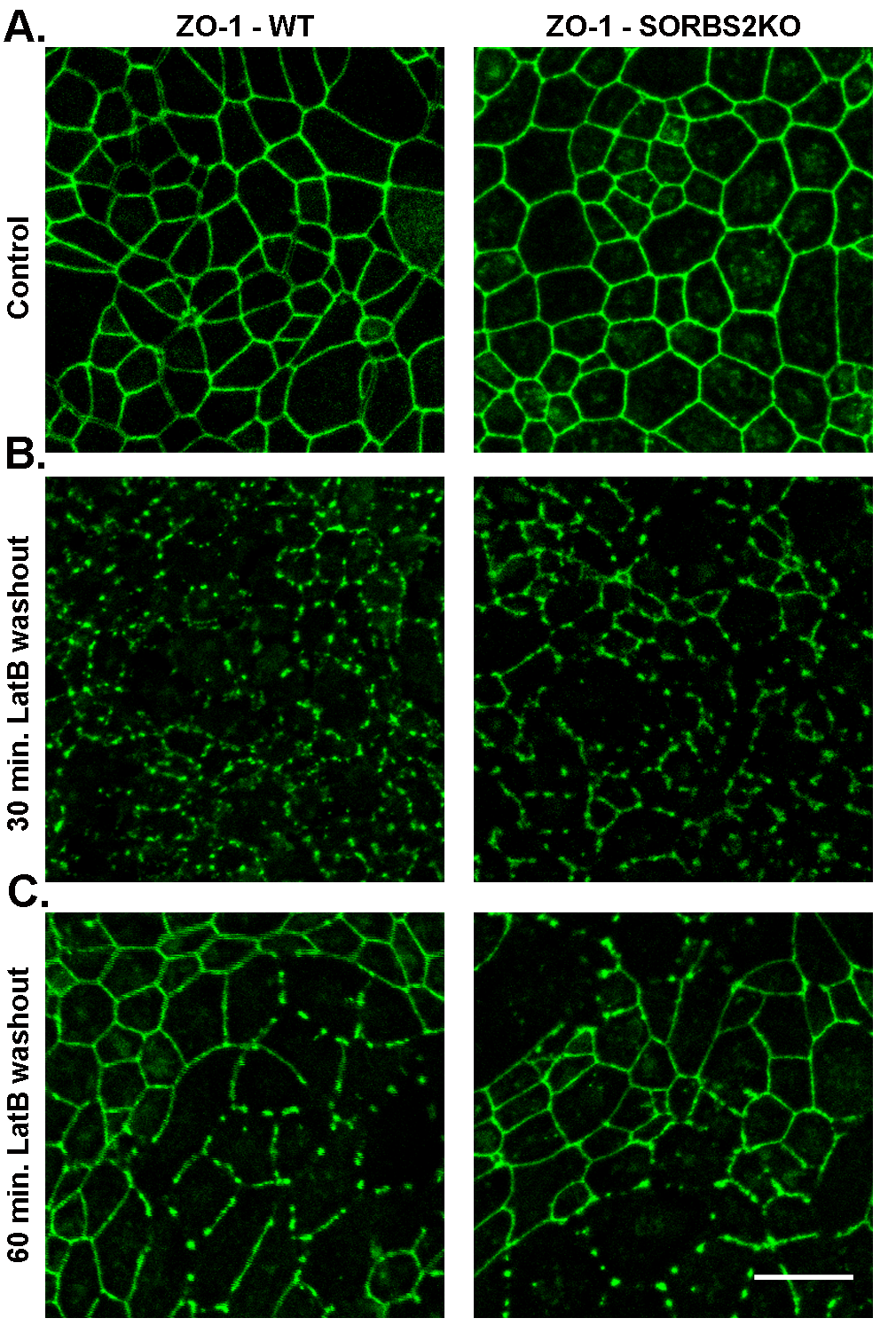
**

Supplement: S2 Fig — Untreated control cells (WT and SORBS2 KO SKco15) or cells exposed to 10uM Latrunculin B for 2 hours, followed by washout and recovery for 30 or 60 minutes, were immunofluorescently labeled with a ZO-1 antibody. Confocal imaging reveled normal ZO-1 localization in control cells (panel A), disrupted ZO-1 localization 30 minutes after Latrunculin B washout (panel B) and partially recovered ZO-1 staining after 60 minutes (panel C). However, there was no difference in recovery between WT and SORBS2 KO cells. 20x objective was used, images are maximum intensity projections (depth: 8.8 μm), scale bar: 20 μm. (DOCX) [file pone.0185448.s002.docx]

**S3 Fig.**

**
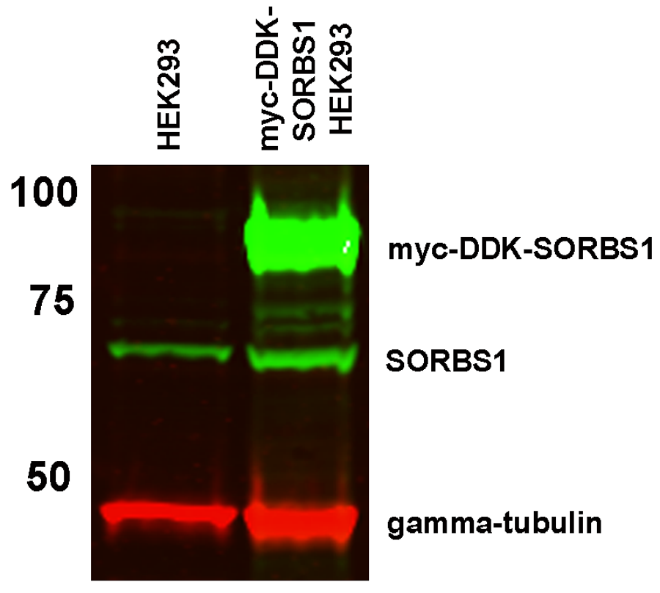
**

Supplement: S3 Fig — To verify antigen recognition and specificity of the SORBS1 antibody we transfected HEK293 cells with myc-DDK-tagged human SORBS1 and immunoblotted the cell lysate in parallel with wild type cell lysate. The SORBS1 antibody used did indeed recognize the SORBS1 fusion protein as well as an endogenous smaller band around 70–75 kDa. There are 12 isoforms of human SORBS1 listed in the Uniprot database ranging in size between 68.7–143 kDa. Two of the 12 isoforms are close to the size identified in wild type HEK293 cells (isoform 4: 76.6 kDa and isoform 7: 68.7 kDa). (DOCX) [file pone.0185448.s003.docx]

**S4 Fig.**

**
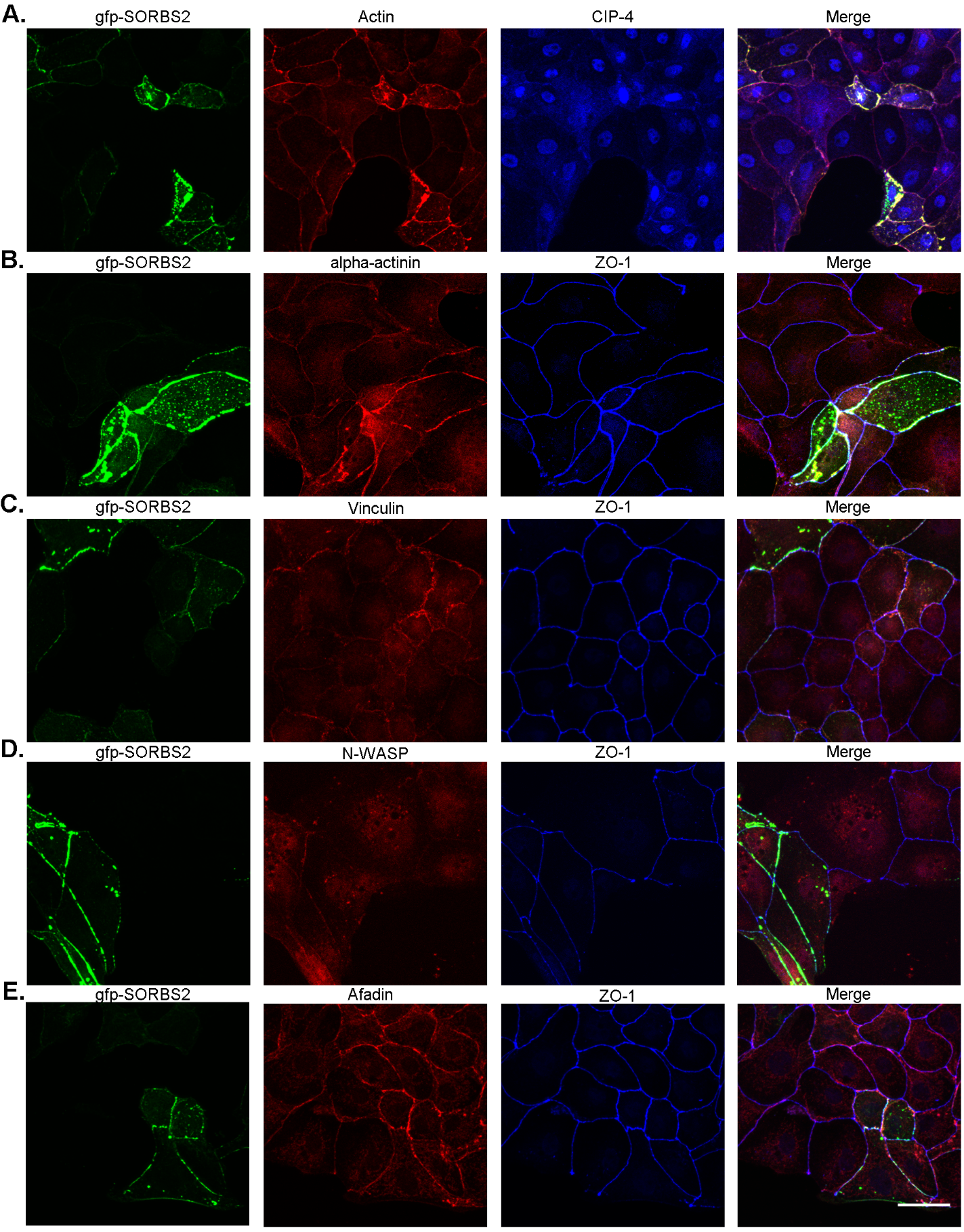
**

Supplement: S4 Fig — As in WT MDCKII cells, expression of GFP-SORBS2 in SORBS2 KO cells is strongly associated with accumulation of actin, alpha-actinin and vinculin (A, B, C) and weakly associated with N-WSAP (D) and possibly CIP4 (A) as shown by confocal immunofluorescence. Afadin accumulation was not associated with GFP-SORBS2 (E). Scale bar: 40 μm. (DOCX) [file pone.0185448.s004.docx]
