# Supplementary material for: Sorbin and SH3 domain-containing protein 2 (SORBS2) is a component of the acto-myosin ring at the apical junctional complex in epithelial cells
Source: PLoS One. 2017 Sep 29;12(9):e0185448. doi: 10.1371/journal.pone.0185448 (PMC5621683; doi:10.1371/journal.pone.0185448)
Supplement: S1 Table — Numbers shown are rank order of the frequency of tagging based on averages of three independent experiments, calculated by normalized peptide-spectrum match divided by observable peptide number as described [8, 9, 30]. ND = not detected. For example, ZO-1 is the #1 protein tagged by biotin ligase fused to the N-terminus of ZO-1 and SORBS2 is #16, higher than the well described TJ protein claudin-4 at position #25. Of the BioID constructs tested SORBS2 is most enriched at the N-terminus of ZO-1. All rank numbers in this table are based on enriched proteins, e.g. we removed all proteins that were three times or less above the biotin ligase alone levels. See references for details [8, 9, 30]. (DOCX) [file pone.0185448.s005.docx]

**S1 Table**

|  | **ZO-1 N-terminal** | **ZO-1 C-terminal** | **Ocln N-terminal** | **Ocln C-terminal** | **Cldn4** | **E-cad** |
| --- | --- | --- | --- | --- | --- | --- |
| **SORBS2** | 16 | ND | 292 | 161 | ND | ND |
| **SORBS1** | 91 | 98 | ND | 344 | ND | 15 |
| **SORBS3** | ND | ND | ND | ND | ND | ND |
| **ZO-1** | 1 | 1 | 19 | 8 | ND | 47 |
| **Ocln** | 6 | ND | 1 | 1 | 22 | 50 |
| **Cldn4** | 25 | ND | 79 | 156 | 33 | ND |
| **E-cad** | ND | ND | ND | ND | ND | 9 |
