# Supplementary material for: Sorbin and SH3 domain-containing protein 2 (SORBS2) is a component of the acto-myosin ring at the apical junctional complex in epithelial cells
Source: PLoS One. 2017 Sep 29;12(9):e0185448. doi: 10.1371/journal.pone.0185448 (PMC5621683; doi:10.1371/journal.pone.0185448)
Supplement: S2 Table — (DOCX) [file pone.0185448.s006.docx]

**S2 Table**

| **SORBS2 CRISPR sg-RNA** | | |  |  |
| --- | --- | --- | --- | --- |
| **Name** | **Species** | **Forward 5'-3'** | **Reverse 5'-3'** | **Exon** |
| 4SC | Dog | CACCTCAAAGCCCCGCACTACCCG | AAACCGGGTAGTGCGGGGCTTTGA | 7 Red=PAM sequence |
| 6SC | Dog | CACCTCAAAGTTCTCCAAACCTAT | AAACATAGGTTTGGAGAACTTTGA | 3 |
| 4SH | Human | CACCTCAAAGCCCCGCATTACCCA | AAACTGGGTAATGCGGGGCTTTGA | 7 |
| 7SH | Human | CACCCAGCATGCGCAGTCTCTGGA | AAACTCCAGAGACTGCGCATGCTG | 7 |
| **DNA sequencing primers for CRISPR KO verification** | | |  |  |
| **Target** | **Species** | **Forward 5'-3'** | **Reverse 5'-3'** |  |
| 4SC | Dog | GCAGATTCCACCGAGGCATA | TCTCTTTCTCCGCCACACAC |  |
| 6SC | Dog | AGATCAGTTGTCCCTGACCCT | TTTAGTTTGGGGTGGGTGTG |  |
| 4SH/7SH | Human | TGGTGAGGCCCTTGTTGGTA | TCACAGCTTCCAATCTCCCG |  |
| **In-Fusion primers** | |  |  |  |
| Forward | 5'-3' | TCTCGAGCTCAAGCTTCTAACACAGGGCGTGATTCTCAGTCA | | Red=vector |
| Reverse | 5'-3' | TAGATCCGGTGGATCCTCACAGCCTCTTGACGTAGTTTCC | |  |
| **qRT-PCR primers** | |  |  |  |
| **Target** | **Species** | **Forward 5'-3'** | **Reverse 5'-3'** |  |
| SORBS1 | Dog | GAGGAGTCTTTGAAACCTTCTCTC | ATTGCTGCCTGGTGCCAACCCGTT |  |
| SORBS2 | Dog | GAGAAATTGCCTGCAAAAGCTGTT | GAGTTTCTCTACATATGAGATCGG |  |
| SORBS3 | Dog | AGTTTGACTTCCAGGCACAG | AGCACCTCCACATAATTAGCAG |  |
| ZO-1 | Dog | Previously published | Previously published |  |
| **Primers for SORBS2 isoform identification (dog)** | | |  |  |
| **Primer pair** | **Species** | **Forward 5'-3'** | **Reverse 5'-3'** | **Size (isoform, mRNA NCBI)** |
| 1 | Dog | AACCTATTGGCTGCAGGGCG | GTACCAGCTCTTTGGTCTGT | 878 (X25), 900 (X23), 1177 (X27) |
| 2 | Dog | Same as above | GACTTCCCAGGTTCATACTC | 900 (X23, X26) |
| 3 | Dog | ACATATTCCTCTCTTGCAGC | Same as above | 900 (X25, X26) |
| 4 | Dog | Same as 1 | TGTTGGAGTTGTTGGGAGAGAG | 850 (X23, X25, X27) |
| 5 | Dog | Same as 3 | CTTGAACATAGTCCAGAGCC | 850 (X23, X25, X26, X27) |
| 6 | Dog | Same as 3 | GAGGACTGATACAAGGAGGC | 500 (X23, X25, X26, X27) |
| **Primers for SORBS2 isoform identification (human)** | | |  |  |
| **Primer pair** | **Species** | **Forward 5'-3'** | **Reverse 5'-3'** | **Size (isoform, UniProt)** |
| 1 | Human | ACCCAGGGATCGGGCCCGTG | TGGGGCTGTTGTCGGAGTGG | 216 (9) 261 (2-5, 8, 12) |
| 2 | Human | GACTGGGATCCTCCAGACA | AAGTCACTGGCCATGCTTGC | 185 (9) 242 (8) 326 (2) 401 (3-5, 12) |
| 3 | Human | GAGCCCTGGTATAAATTCTT | Same as above | 176 (2) |
| 4 | Human | TCTCCAAACCTATTGGCTGC | CATCCACGGGCCCGATCCCT | 457 (9, 12) |
| 5 | Human | CCACCCCTGCGGCCCACTCA | Same as above | 303 (3) |
| 6 | Human | Same as above | TGGGGCTGTTGTCGGAGTGG | 540 (3) |
